# Epigallocatechin Gallate/Layered Double Hydroxide Nanohybrids: Preparation, Characterization, and *In Vitro* Anti-Tumor Study

**DOI:** 10.1371/journal.pone.0136530

**Published:** 2015-08-28

**Authors:** Seyedeh Sara Shafiei, Mehran Solati-Hashjin, Ali Samadikuchaksaraei, Reza Kalantarinejad, Mitra Asadi-Eydivand, Noor Azuan Abu Osman

**Affiliations:** 1 Department of Stem cell and Regenerative medicine, Institute of Medical Biotechnology, National Institute of Genetic Engineering and Biotechnology, Tehran 14965/161, Iran; 2 Biomaterials Center of Excellence, Amirkabir University of Technology, Tehran 15914, Iran; 3 Department of Biomedical Engineering, Faculty of Engineering, University of Malaya, Kuala Lumpur 50603, Malaysia; 4 Cellular and Molecular Research Center, Iran University of Medical Sciences, Tehran, Iran; 5 Department of Tissue Engineering and Regenerative Medicine, Faculty of Advanced Technologies in Medicine, Iran University of Medical Sciences, Tehran, Iran; 6 Department of Medical Biotechnology, Faculty of Allied Medicine, Iran University of Medical Sciences, Tehran, Iran; 7 Converging Technology Research Center, Hamgara, Tehran, Iran; Winship Cancer Institute of Emory University, UNITED STATES

## Abstract

In recent years, nanotechnology in merging with biotechnology has been employed in the area of cancer management to overcome the challenges of chemopreventive strategies in order to gain promising results. Since most biological processes occur in nano scale, nanoparticles can act as carriers of certain drugs or agents to deliver it to specific cells or targets. In this study, we intercalated Epigallocatechin-3-Gallate (EGCG), the most abundant polyphenol in green tea, into Ca/Al-NO_3_ Layered double hydroxide (LDH) nanoparticles, and evaluated its efficacy compared to EGCG alone on PC3 cell line. The EGCG loaded LDH nanohybrids were characterized by X-ray diffraction, Fourier transform infrared spectroscopy, transmission electron microscopy (TEM) and nanosizer analyses. The anticancer activity of the EGCG-loaded LDH was investigated in prostate cancer cell line (PC3) while the release behavior of EGCG from LDH was observed at pH 7.45 and 4.25. Besides enhancing of apoptotic activity of EGCG, the results showed that intercalation of EGCG into LDH can improve the anti- tumor activity of EGCG over 5-fold dose advantages in *in-vitro* system. Subsequently, the *in-vitro* release data showed that EGCG-loaded LDH had longer release duration compared to physical mixture, and the mechanism of diffusion through the particle was rate-limiting step. Acidic attack was responsible for faster release of EGCG molecules from LDH at pH of 4.25 compared to pH of 7.4. The results showed that Ca/Al-LDH nanoparticles could be considered as an effective inorganic host matrix for the delivery of EGCG to PC3 cells with controlled release properties.

## Introduction

Cancer is one of the most fatal forms of illnesses all over the world, and it is foreseen that cancer will be the most frequent disease by 2020 [1]. Specialists still consider chemotherapy as the main treatment for many cancers in advanced levels. On the other hand, chemotherapy usually is associated with severe side effects such as anemia, exhaustion, headache, nausea, hair loss, vomiting, and loss of appetite. In the initial stages of cancer, however, one promising approach for inhibition or decelerating the development of carcinogenesis is chemoprevention. This term is defined as the use of natural non-toxic agents for dealing with different types of cancer [2].

Among chemopreventive agents, flavonoids are well known for their multi-directional biological activities. Flavonoids are low-molecular weight polyphenols present in plant foods, fruits, herbs and tea, which are divided into categories based on their structures. Flavan-3-ol, also known as catechin, is the most abundant compound in green tea (Camellia sinensis), accounting for 30–40% of its dry weight. The major catechins extracted from tea leaves include (-)-epigallocatechin (EGC), (-)-epicatechin-3-gallate (ECG) and (-)-epicatechin (EC) and (-)-epigallocatechin-3-gallate (EGCG).Among these catechins, EGCG, the most biologically active ingredient of green tea, has been shown to have chemopreventive property in several cell culture media and preclinical studies [3].

Several reports [4], [5] have shown a remarkable reduction in the prostate cancer cell number in response to treatment with EGCG. The major chemopreventive activities of EGCG include inhibition of the activity of many protein kinases, inhibiting the activity of the epidermal growth factor receptor (EGFR) in prostate cancer, inhibition of cell proliferation, induction of apoptosis, regulation of cell cycle arrest, interference of receptor binding, and suppression of angiogenesis.

Although chemoprevention showed promising outcomes in preclinical works, including decreasing side effects, its efficiency has met with limited success. The primary limit is inefficient delivery of chemopreventive agent to the target site. This can be related to poor systemic bioavailability of the agent and its instability in the living environment. Therefore, new approaches are necessary to increase the bioavailability of these potentially helpful agents to reach the best possible therapeutic response [6].

Recently, nanotechnology in combination with biotechnology has been employed in cancer management area to improve the outcome of chemopreventive approaches. Since biological processes mostly occur in nano-scale, nanoparticles can be used as potential carriers to deliver certain drugs or agents to specific cells or targets in the human body [7].

Several reports have recently been published on studies of effectiveness of EGCG/nanoparticles in treatment of cancers. Wu et al. [6] reported EGCG in conjunction with gold-nanoparticles is more efficient than free EGCG in suppression of tumor. Siddiqui et al. [8] showed nanochemoprevention using biocompatible PLA-PEG nanoparticles enhanced bioavailability and therapeutic effectiveness of EGCG. Furthermore, nano-EGCG exhibits superior effects such as dose advantages over nonencapsulated EGCG. In another research, Fang et al. [9] discovered that the incorporation of deoxycholic acid into the liposomes in the presence of 15% ethanol greatly increased EGCG uptake by the tumor cells. He suggested that liposome delivery systems could serve as effective EGCG carriers providing more stability inside vesicles.

Inorganic nanoparticles have lately been attracting attention. Layered double hydroxides (LDHs) are a class of synthetic anionic clays whose structure can be described as brucite (Mg (OH) _2_)-like layers that some of the divalent cations have been replaced by trivalent ions, resulting in positively charged sheets. The natural mineral hydrotalcite [Mg_6_Al_2_(OH)_16_CO_3_·4H_2_O] is classified in this group. Most LDHs have the generic formula of [M_1−x_^2+^M_x_^3+^(OH)_2_][A_x/n_^n−^]·mH_2_O, where M^2+^ and M^3+^ are di- and tri-valent cations, respectively. A^n−^ is the exchangeable interlayer anion, m is the number of interlayer water, and x = M^3+^/(M^2+^+M^3+^) represents charge density of LDH [10]. Due to many versatile properties, including low toxicity, high reserving capacity, and enhanced cellular uptake behavior, LDH is found to be an effective delivery carrier for a variety of biologically active compounds such as anti-cancer drugs[11–14]. Choy et al. [15] and Wang et al. [16] reported the intercalation of methotrexate (MTX) and 5-Fu with LDH, which leads to remarkable increase in drug delivery effectiveness and suppression of tumor. In a more recent study, Gu et al. [17] showed LDHs has a great potential to co-deliver siRNA and an anticancer drug to cancerous cells leading to enhance cancer treatment.

In this study, an EGCG-LDH nanohybrid was designed and developed as a potential new cancer chemopreventive agent. Epigallocatechin gallate (EGCG) of green tea was successfully intercalated into wet chemically synthesized Ca-Al-LDH by ion-exchange techniques. In order to understand whether these nanoparticles show an enhanced chemotherapeutic effectiveness, we evaluated the anti-tumor activity of EGCG-loaded LDH nanohybrids in PC3 prostate cancer cell line *in-vitro*, and compared the results with that of free EGCG.

## Materials and Methods

### Materials

Inorganic reagents, including calcium nitrate (Ca(NO_3_)_2_.6H_2_O, Merck 102121), aluminum nitrate (Al(NO_3_)_3_.9H_2_O, Merck 101063) and Sodium Hydroxide (NaOH, Merck 106462) were used without further purification. Deionized water was decarbonated by boiling and bubbling N_2_ before being used in all synthesis steps. Epigallocatechin gallate, hereafter called EGCG, was purchased from Enzo Life Science—Alexis (USA). Other chemicals and solvents used for the synthesis and tests were of analytical grade (AR) and used without further purification. Annexin-V-FLUOS Staining Kit (Cat. No. 1185877700150 tests) was purchased from Roche (Germany). PC3 cells were obtained from National Cell bank of Iran (NCBI), Pasteur Institute (Tehran, Iran).

### Preparation of Ca/Al-nitrate LDHs

LDH samples were synthesized by a co-precipitation method based on the protocol of Miyata [10]. Typically, An aqueous solution (100 mL) containing NaOH (0.6 mol) was added drop wise to a solution (150 mL) containing Ca(NO_3_)_2_.6H_2_O (0.2 mol) and Al(NO_3_)_3_.9H_2_O (0.1 mol) (Ca/Al molar ratio = 2.0) with vigorous stirring. pH was adjusted to 10.0. In order to avoid atmospheric CO_2_contamination; all processes were performed under nitrogen ambient. The resulting gel-like mixture was aged for 18 h at room temperature (25°C). Then centrifuged and washed several times with deionized water until an almost neutral pH was reached. After a final centrifugation step (4500 rpm for 5 min, Hettich, Universal 320 R), a known amount of resulting precipitate was re-suspended in 50 mL deionized water and transferred to a Teflon-coated stainless steel autoclave for a hydrothermal post treatment. The autoclave was then sealed and heated in an electric oven (Memmert UNB 400) at 120°C for 4h, before naturally cooling down to room temperature. A certain amount of obtained suspension was taken for further investigations. The remaining suspension was freeze dried to obtain a white powder. We reported the details of the synthesis process in our previous paper [18].

### Preparation of EGCG–LDHs nanohybrids

The EGCG intercalated nitrate LDH was prepared by the ion exchange method. The intercalation reaction was performed by mixing a solution of excess amount of EGCG (NO_3_^-^/EGCG molar ratio = 1/2), and aqueous suspension containing 0.1 g Ca/Al-NO_3_–LDHs, respectively. The mixture was incubated at 37°C for 24 h in a rotary shaker, followed by centrifugation for 5 min (18,000×g).The supernatant (representing the unloaded concentration of EGCG) was analyzed using UV-Vis Spectrophotometer (SolidSpec-3700 UV-VIS-NIR, Shimadzu, Japan). The amount of EGCG, hereafter EGCG-LDH, was calculated by subtracting the remained amount from the total amount of drug added.

### Characterization

Identification of the crystalline phase of synthesized LDH powder was performed using a Bruker D4 Endeavor diffractometer (Germany). X-ray diffraction (XRD) patterns were recorded over the 2Ѳ range of 5–70° at a scan rate of 2° min^-1^ with a monochromatic Cu K_α_ radiation (γ = 1.5406 A°. 40 kV, 30mA, 0.02° step scan).

Fourier transform infrared spectrum was obtained on a Bruker IFS 48 spectrometer (Germany). 2 mg of LDH powder compacted with 200 mg of potassium bromide under a hydraulic pressure. The spectrum was recorded in the 4000–400 cm^-1^ region with 2 cm^-1^resolution averaging 100 scans. The average particle size (z average size) and size distribution were recorded by Nanosizer Nano ZS, Malvern Instruments (UK).

The morphology of the synthesized LDH particles was characterized by transmission electron microscopy (TEM) using a Philips EM208 (Netherlands) at an acceleration voltage of 200 kV. For sample preparation, the freshly prepared LDH nanoparticles were dispersed in alcohol with ultrasonication for 30 min, and then, a droplet was dropped on a copper grid coated with amorphous carbon film.

### Cell culture

Prostate cancer PC3 cell line was used in this study. Cells were cultivated in L-glutamine containing RPMI-1640 medium supplemented with 10% fetal bovine serum (Gibco, USA) and 1% penicillin/streptomycin in T75 cm^2^ flasks at 37°C in a humidified atmosphere with 5% CO_2_. When cultured cells reached 70–80% confluence, they were seeded in a round bottom 96 well plate at density of 10^3^ to 10^4^ 24h prior to the performance of the subsequent assays.

### Cell viability

We used a cell viability test similar to the work of Qin et al. (2010) to estimate the suppression effect of LDH, EGCG and EGCG-LDH against PC3 cells. PC3 Cells were cultured in RPMI-1640 and 10% fetal bovine serum was added. The culture medium then was incubated at 37°C in a 5% CO_2_ humid atmosphere. Certain amount of cells (100μl) were cultured at a density of almost 10^4^ cells per well in a 96-well plate, and afterward were incubated in the same incubation condition. To each group (triplicate wells) was added definite quantity of LDH, EGCG and EGCG-LDH and the incubation was continued for 24, 48 and 72 h. Then, 20 μl of MTT solution (5 mg/ml) was added to each well and the cultures were incubated again for 4 h at 37°C. Finally, the culture medium was removed, and formazan crystals within cells were solubilized using 200 μl of DMSO for 10 min in the dark.

The absorbance was measured by ELISA microplate reader at 570 nm and reference filter at 630 nm. Since reduction of MTT only arises in metabolically active cells, the level of activity can be considered as a measure of viability. Cell viability was calculated with the following formula:

Cell viability(%)=OD590(sample)−OD590(blank)/OD590(control)−OD590(blank)×100

### Detection of apoptosis

The Annexin V–FLUOS staining assay for apoptosis was performed. PC3 cells were incubated with or without EGCG, LDH and EGCG-LDH for predefined time. Briefly, The cells were grown on cell culture slides and then treated with EGCG alone, LDH, and EGCG-LDH formulations. Then cells were covered by 100 μl of Annexin-V-FLUOS labeling solution containing diluted solution of Annexin V-FITC and Propidium Iodide. Afterward, the cells were incubated with Annexin V-FLUOS labeling reagent for 10 min in the dark and then identified using fluorescence microscopy (excitation at 450–500 nm and detection at 515–565 nm). Cells with green fluorescence were scored as apoptotic. Necrotic cells take up propidium iodide and stain red, while apoptotic cells stain green only. For quantitation, apoptotic cells were counted in randomly selected fields and represented as mean apoptotic cells (apoptotic index (AI)).

### Colony formation assay

Colony formation assay which is an *in-vitro* cell survival assay was used to study the effectiveness of the anti cancer drugs against cancer cell proliferation. PC3 cells were cultured in RPMI1640 with 10% FBS and 1% penicillin/streptomycin, seeded in tissue culture dishes at density of (8 × 10^2^) and incubated at 37°C, prior to treatments. LDH, EGCG and EGCG-LDH were added to each group after 24 h and incubated at 37°C for a total incubation period of 14 days. The medium was removed from the cells, washed with PBS and Colonies were fixed with 10% buffered formalin stained with hematoxylin and eosin. The excess stain was washed with water and then air-dried. Finally, the colonies (>50 cells) were counted using a light microscope.

### In-vitro release study

0.05g of EGCG–LDHs was dispersed in 250 ml phosphate buffered solution (PBS) with pH = 7.45 followed by incubation in a water bath at 37°C with gentle shaking. Aliquots of 1 mL were extracted at given time intervals, centrifuged at 12000 rpm and replaced by equal volumes of fresh PBS. The amount of released EGCG was determined by a UV–Vis spectrophotometer with an absorbance wavelength of 517 nm. The runs were made in triplicate and the results were recorded as an average. The same release study procedure was performed in PBS solution with pH = 4.25 (adjusting by NaOH and HCl solutions 0.1 M). The release profile obtained from the formulation EGCG–LDHs was compared with the physical mixture of LDH and EGCG.

The kinetic model for EGCG ions released from the EGCG–LDHs was analyzed by fitting the released data with the kinetic models.

### Statistical analysis

Data were represented as means ± standard deviation. Statistical significance was assessed by one-way Anova multi-comparison. Values of P < 0.05 were accepted statistically significant.

## Results and Discussion

### Characteristics of LDH and EGCG-LDH

**Fig 1:**
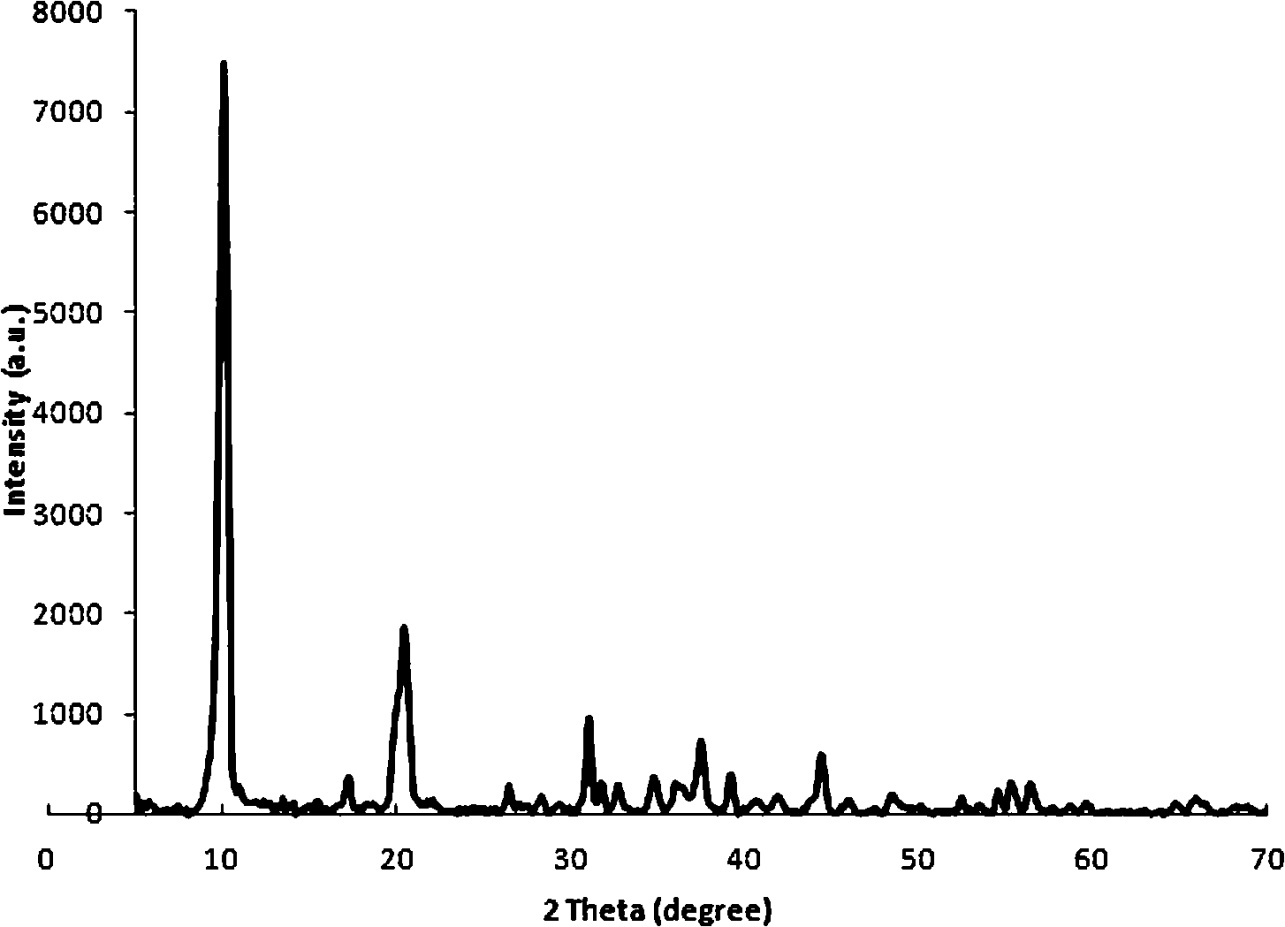
X-ray diffraction patterns for LDH-NO_3_ particles.

The XRD patterns for the synthesized LDH were shown in Fig 1. The LDH exhibited the Bragg reflections of basal planes. The series of (00l) peaks were symmetric at low 2Ѳ angles (002, 004) but broad and asymmetric at high 2Ѳ angles (110). From the lattice parameters of samples, the value of the basal spacing (0.86 nm) corresponded to the sum of the thickness of the hydroxyl layer ([Ca_2_Al(OH)_6_], 0.48 nm) and of the interlayer anions (nitrates), which was 0.38 nm. This was in agreement with the previous reported value for the basal spacing of LDH, including the planar orientation of nitrates and water molecules within the interlayer space of LDH [19].

**Fig 2:**
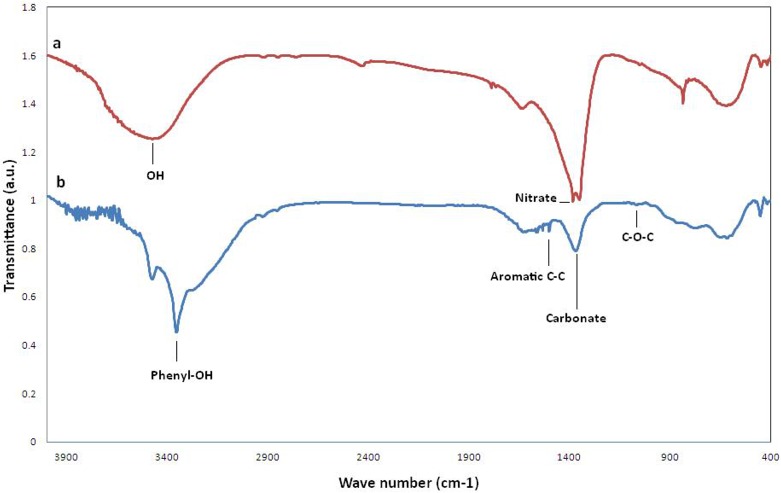
Fourier transform infrared spectroscopy spectrum of a) LDH-NO3 and b) EGCG-LDH.

The FT-IR spectra of EGCG-LDH and LDH were included in Fig 2. Intercalation of EGCG into LDH was confirmed by the presence of the 3600–3150 cm^-1^ band which is the characteristic peak of phenyl—OH groups abundant in EGCG (Fig 2B). Furthermore, the absence of the band at 1384 cm^-1^(the characteristic band of NO_3_) indicated that the interlayer nitrate ions were successfully exchanged by EGCG ions (Fig 2C).

Additionally, the characteristic bands of EGCG were found at 1500 cm^-1^ for aromatic C-C stretching and at 823 cm^-1^ for C–H alkenes. The bands below 1000cm^-1^ were assigned to the lattice vibration mode of M–O and M–O–M (Ca and Al) in the LDH matrices and the presence of a few co-intercalated CO_2_^-3^ anions were also confirmed by the band at 1365cm^-1^.

**Fig 3:**
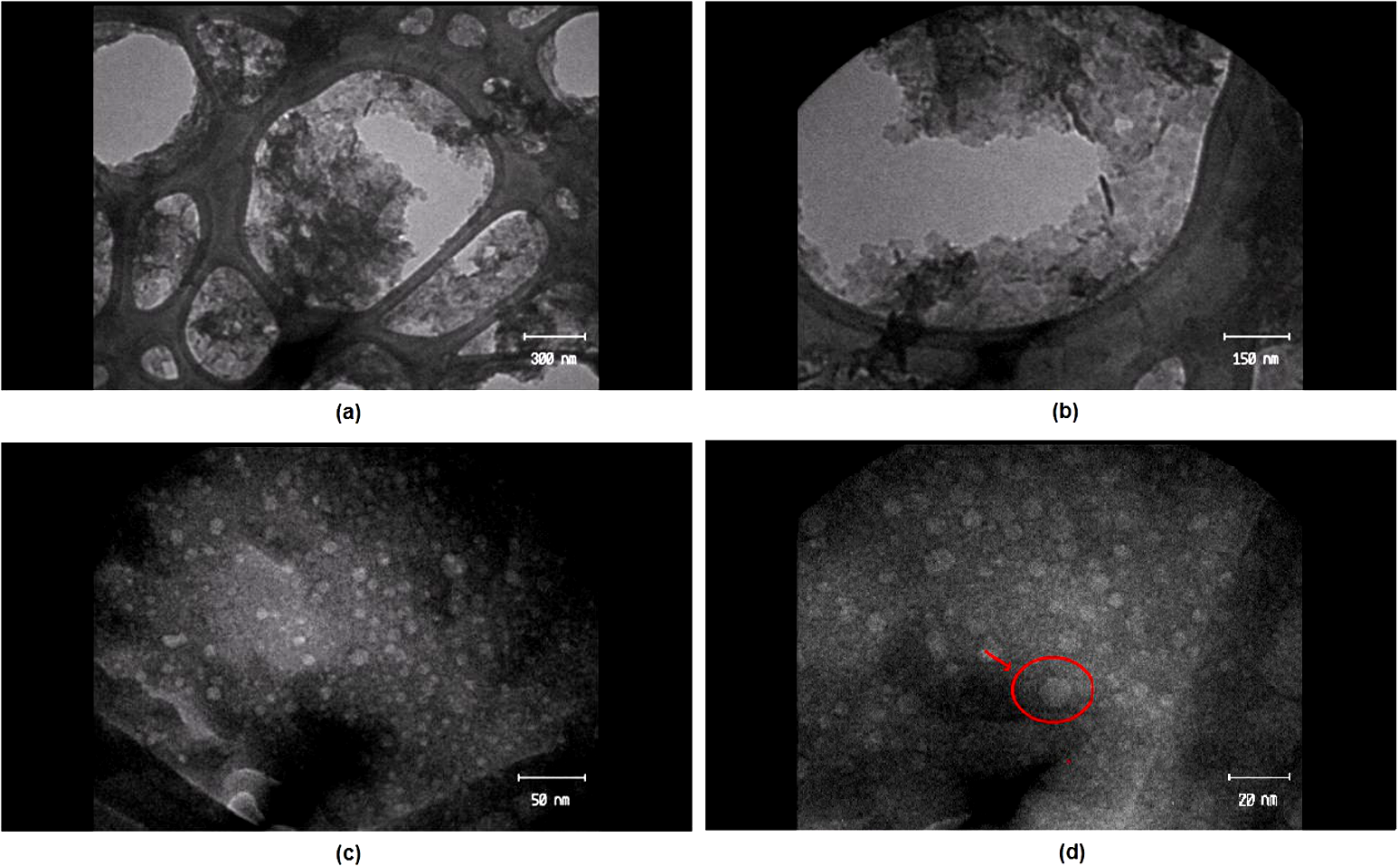
Images (TEM) of EGCG-LDH nanohybrids with scale bars of a) 300nm, b) 150nm, c) 50nm, and d) 20nm.

**Fig 4:**
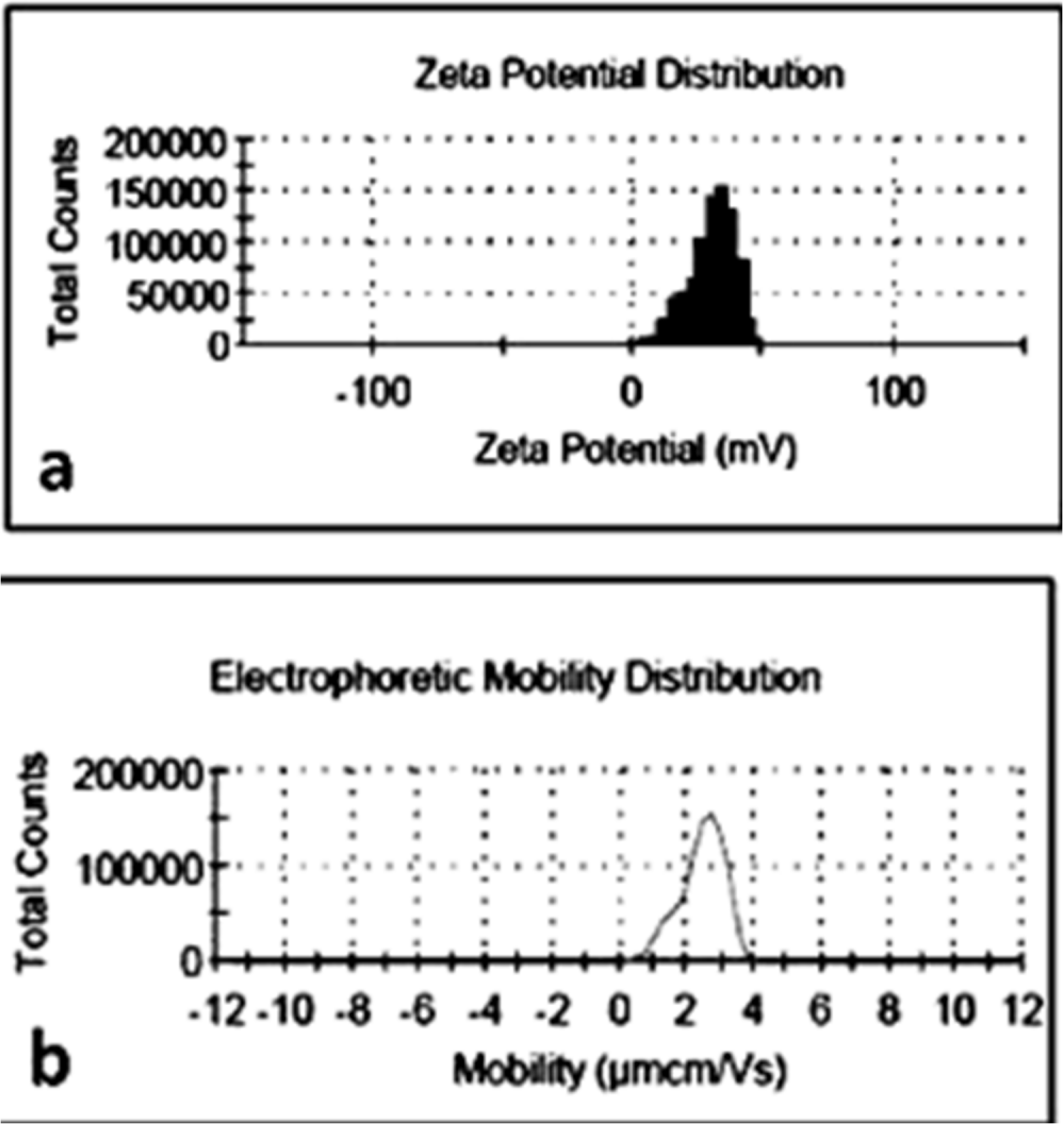
a) zeta potential and b) electrophoretic mobility spectra of EGCG-LDH nanohybrids.

From the TEM images shown in Fig 3, the EGCG-LDH nano-flakes were very properly separated and well shaped in a hexagonal form with a lateral size of 10 nm (see the arrow in Fig 3D). Furthermore, as shown in Fig 4, the zeta potential and electrophoretic mobility of nano-hybrid were measured as +30.6 mV and 2.426 μmcm/Vs, respectively. The relative high surface charge of LDH made it more likely to interact with negatively charged cell membranes and be taken up by the cells easily.

### In vitro anti-tumor activity of EGCG-LDH

**Fig 5:**
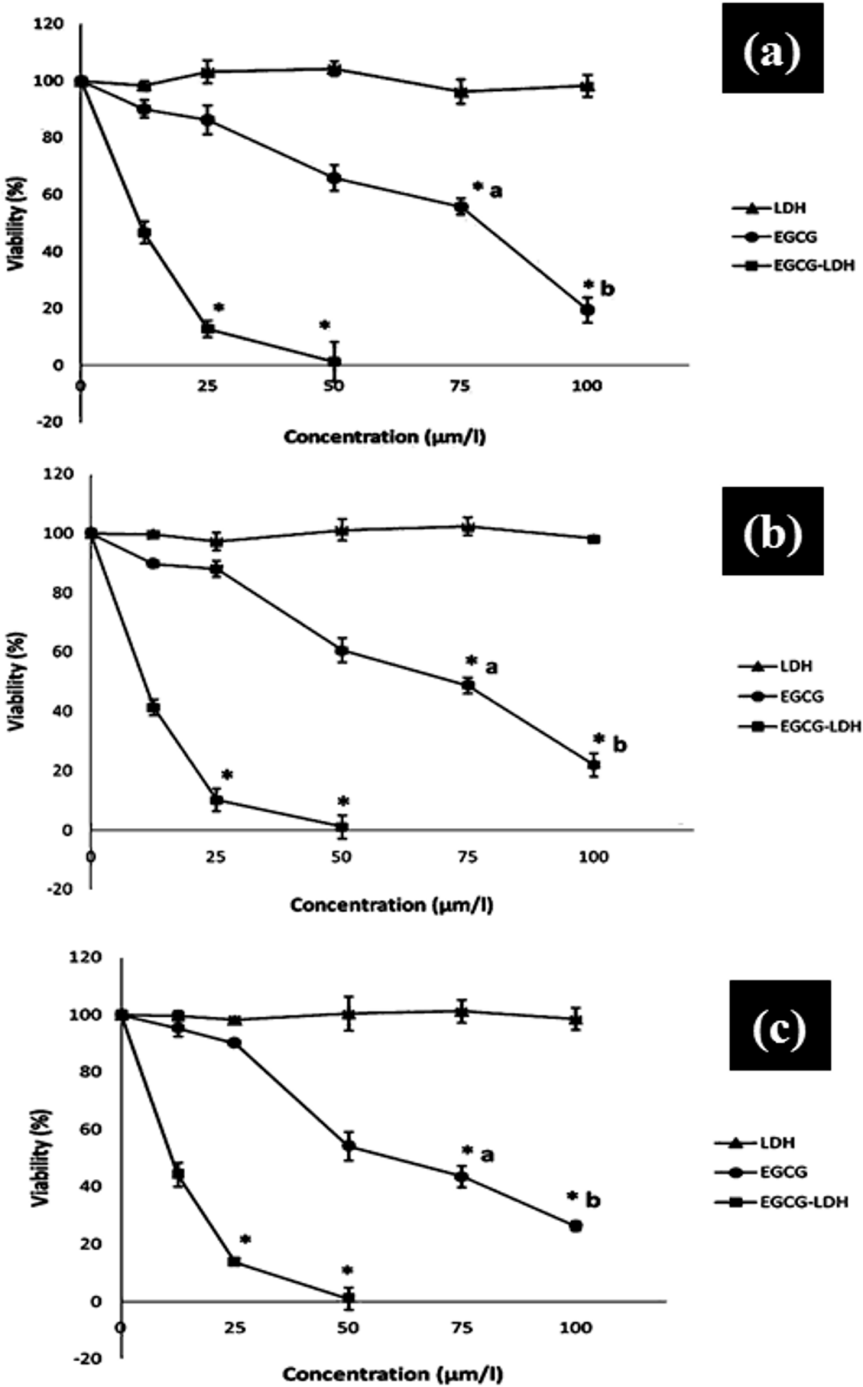
PC3 cells were treated with void LDH, EGCG, and EGCG-LDH for a) 24 h, b) 48h and 72h. Cell growth was determined by MTT assay. Points, mean of three separate experiments wherein each treatment was repeated in 10 wells; bars, SE. *, P < 0.001 compared with the vehicle-treated control. p<0.05, *a vs. EGCG-LDH-25 and *b vs. EGCG-LDH-50.

We investigated the effects of EGCG, EGCG-LDH and LDH in PC3 prostate carcinoma cell line. In the first part of this study, the cytotoxic activities of samples were determined using an MTT assay. This colorimetric assay was based on the reduction of a soluble tetrazolium salt, by mitochondrial dehydrogenase activity of viable cells, into a soluble colored formazan product that could be measured by spectrophotometer after dissolution. The IC50 value was used to quantify cytotoxicity. The viability of PC3 cells versus time is shown in Fig 5. EGCG and EGCG-LDH suppressed the cell viability of tumor cells, while LDH itself had no significant effect. The EGCG and EGCG-LDH were both able to exert cytotoxic effects in the PC3 cell line tested in a dose-dependent manner.

The effectiveness of EGCG-LDH was compared with non-intercalated EGCG on viability in human PC3 cells. Interestingly, EGCG-LDH had a higher tumor suppression efficiency compared to EGCG at predefined incubation times. The results showed that EGCG-LDH had a better effect with approximately 5-fold dose advantage in the sense that the cell viability of EGCG-LDH was much lower than that of EGCG only after 24h treatment. As shown in Table 1, the half maximal inhibitory concentration (IC50 value) of EGCG (at 24 h post treatment) was found to be 16.66 μmol/L compared with 69.73μmol/L of non-intercalated EGCG. These results were indelible even at 48 and 72 h post-treatment (Fig 5B and 5C). As indicated by the MTT assay analysis results, LDH nanoparticles did not show a cytotoxic effect even after 72h of exposure. In contrast, EGCG-LDH induced cytotoxic activity in prostate carcinoma, PC3 cell line.

**Table 1:** Half maximal inhibitory concentration (IC50 value) of EGCG, EGCG-LDH after 24, 48 and 72h exposure to cells.

IC50 (μm/L)		
Time	EGCG	EGCG-LDH
**24h**	69.73	16.66
**48h**	67.49	15.47
**72h**	66.83	16.33

These findings suggested that there was no premature decomposition of EGCG molecules in the LDH hybrid system and they could effectively reach the PC3 cell membrane. This was an indication of stabilization of the EGCG molecules inside the LDH structure after intercalation. In addition, the results disclosed that it was possible for that EGCG-LDH nanoparticles to move into the cells via endocytosis. Thus, EGCG-LDH could be more quickly taken up by PC3 cells and the controlled release of EGCG could also affect the exposure time allowed for more tumor cells to be affected in order to increase the drug efficiency.

### Apoptosis detection

The Annexin V–FLUOS staining assay for apoptosis was performed, as described in Materials and Method section. Both untreated and treated PC3 cells were sequentially treated with Annexin V–propidium iodide and apoptotic cells were evaluated by a fluorescence microscope. Random microscopic fields were analyzed to assess the fraction of annexin V+ cells for each sample. As a result, no significant apoptosis was observed in the cell treated with void LDH and control group.

Untreated cells were represented as the control, i.e., the PC3 cell line cultured in complete media for 48 hours. The control cells showed that only 1.89% of these cell deaths produced a typical morphological apoptotic feature (Table 2). In addition, void LDHs showed only 2.86% of apoptotic death. In contrast, the AI percentage of PC3 cells increased significantly when the PC3 cell line was treated with 12.5 μM of EGCG compared to the control. The AI percentage continued to increase substantially as well when the cell line was treated with EGCG at 75 μM compared to the control.

**Table 2:** Percent of apoptotic cells. annexin V- propidium were counted in the field of view, which included 1000 cells, using an optical microscope. (Magnification, ×400).

Percent of apoptotic cells (%)				
Concentration (μm)	Control	Void LDH	Non-intercalated EGCG	EGCG-LDH
0	1.89	×	×	×
12.5	×	×	8.76	60.39
25	×	×	11.04	71.15
75	×	2.89	72.89	78.56

**Fig 6:**
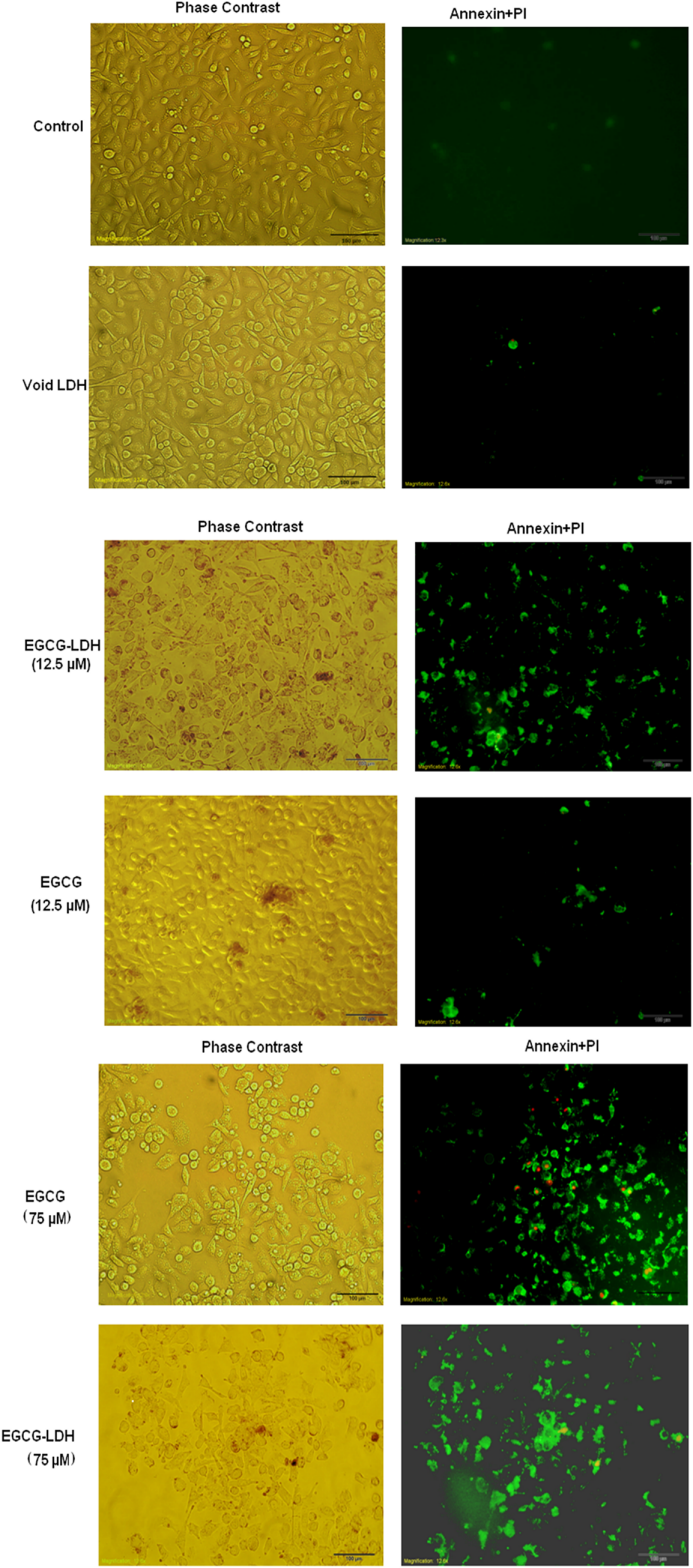
Apoptosis detection. PC3 cells were grown on cell culture slides and treated with EGCG and EGCG-LDH and void LDH for 48 h. Apoptosis was determined, as detailed in Materials and Methods. Representative photomicrographs from each treatment group showing induction of apoptosis (green fluorescence). Data are from experiment repeated thrice with similar results.

These observations indicated that the apoptotic activity was gradually increased when the concentration of EGCG exposed to PC3 cells increased (Fig 6). On the other hand, nanoparticles showed the same trend by increasing the concentration from 12.5 to 25 μM by which the apoptotic activity was enhanced. As a result, It was found that 25 μmol/L of EGCG-LDH caused approximately 71.15% apoptosis in PC3 cells. To achieve the similar extent of apoptosis, 75 μmol/L of non-intercalated EGCG was required, thereby providing a remarkable dose advantage. Moreover, 12.5 μmol/L of EGCG had no obvious effects on the apoptosis of PC3, while at the same concentration EGCG-LDH caused approximately 60.39% apoptosis in the cells. Overall, we observed enhanced apoptosis of PC3 cells treated with EGCG-LDH compared with non-encapsulated EGCG.

Typically, the staining with Annexin V was associated with a dye such as propidium iodide (PI) for recognition of early and late apoptotic cells. Viable cells with healthy membranes excluded PI, while the dead and damaged cells took up PI. Therefore, cells that were considered viable are both Annexin V and PI negative, whereas cells that were in early apoptosis were Annexin V positive and PI negative. Cells that were in late apoptosis or necrosis were both Annexin V and PI positive. Therefore, this assay did not determine whether cells have undergone apoptotic death or have died as a result of a necrotic pathway because they also stained with both Annexin V and PI. Nevertheless, the presence of cells with these three stages within a mixed cell population, suggested a time-dependent apoptosis process.

**Fig 7:**
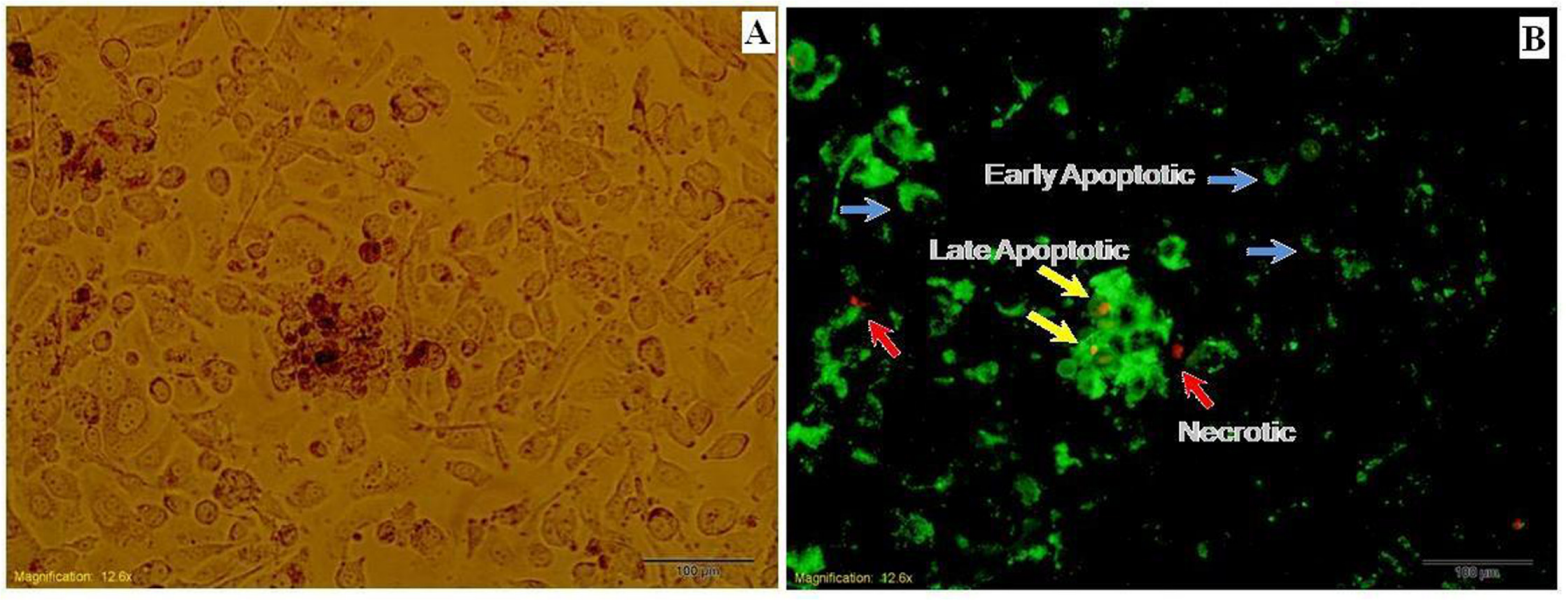
PC3 cells exhibited early apoptotic (indicated by blue arrows) and necrotic and late apoptotic (indicated by yellow and red arrows, respectively) cell death after 48 h post exposure to 25 μM EGCG-LDH.

As shown in Fig 7, the result of annexin-V-PI staining of PC3 cells treated with 25 μmol/L EGCG-LDH exhibited early apoptotic (stained green, indicated by blue arrows), late apoptotic (stained orange, indicated by yellow arrows), and necrosis (stained red, indicated by red arrows) cell death after 48 h post exposure. Altogether, the observations confirmed the presence of cells with these three stages in time-dependant apoptosis process.

### Colony formation assay

**Fig 8:**
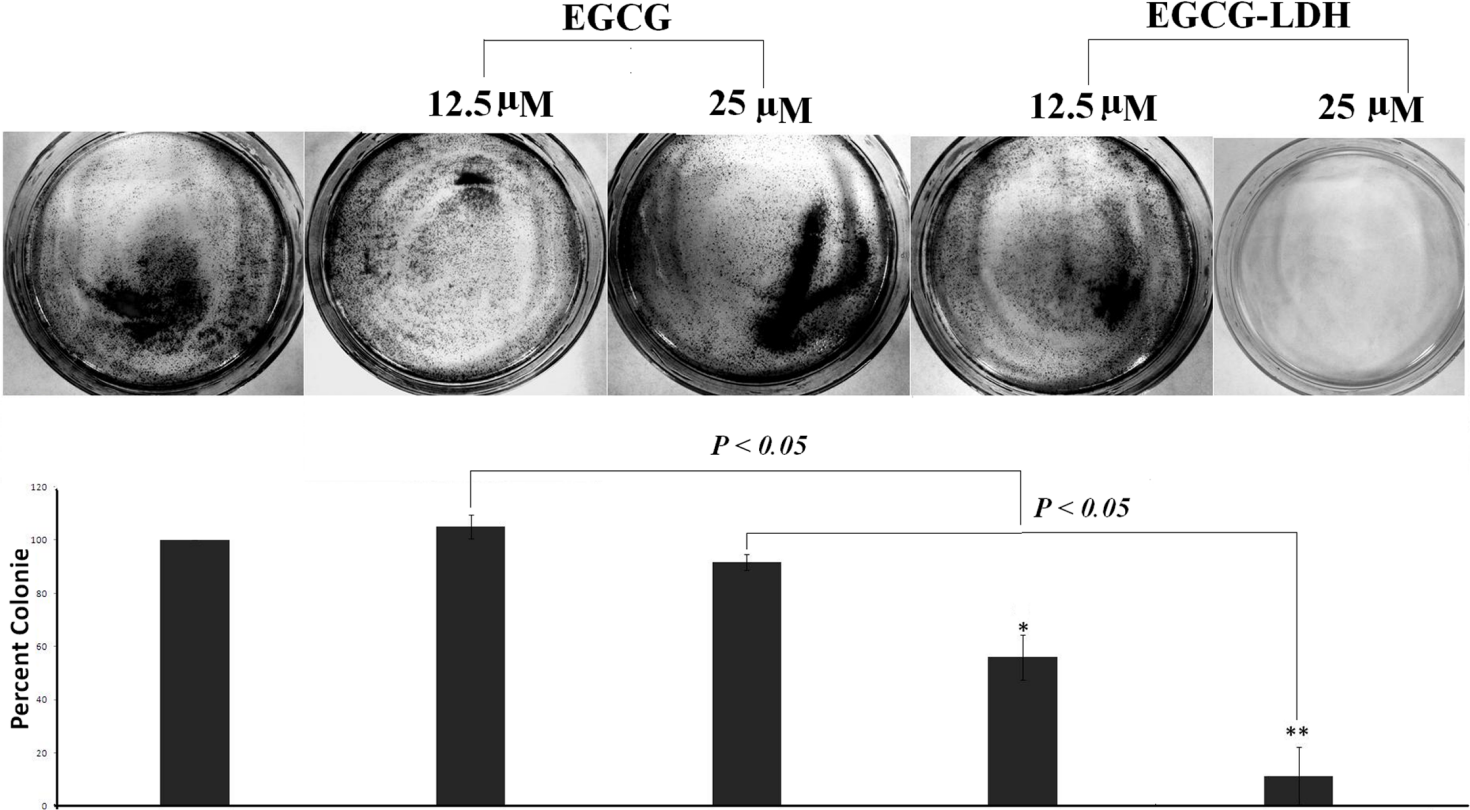
Colony formation. PC3 cells were treated with EGCG and EGCG-LDH with different concentrations, and the plates were observed for colonies, counted, and plotted as a bar graph. Bars, SE. *, P < 0.05; **, P < 0.01 compared with the vehicle-treated controls. The results are from a representative experiment repeated two times with similar results.

The long-term suppression effect of EGCG and EGCG-LDH was examined with the colony formation assay. In this experiment, only a fraction of seeded cells had the potential to produce colonies after treatments. As showed in Fig 8, PC3 colony forming reduced significantly when the concentrations of EGCG-LDH increased from 12.5 μmol/L to 25 μmol/L. Using colony formation assay, EGCG-LDH was found to show remarkable colony formation suppression, providing a significant effect at 25 μmol/L compared with 25 μmol/L of non-intercalated EGCG.

### Sustained release of EGCG

**Fig 9:**
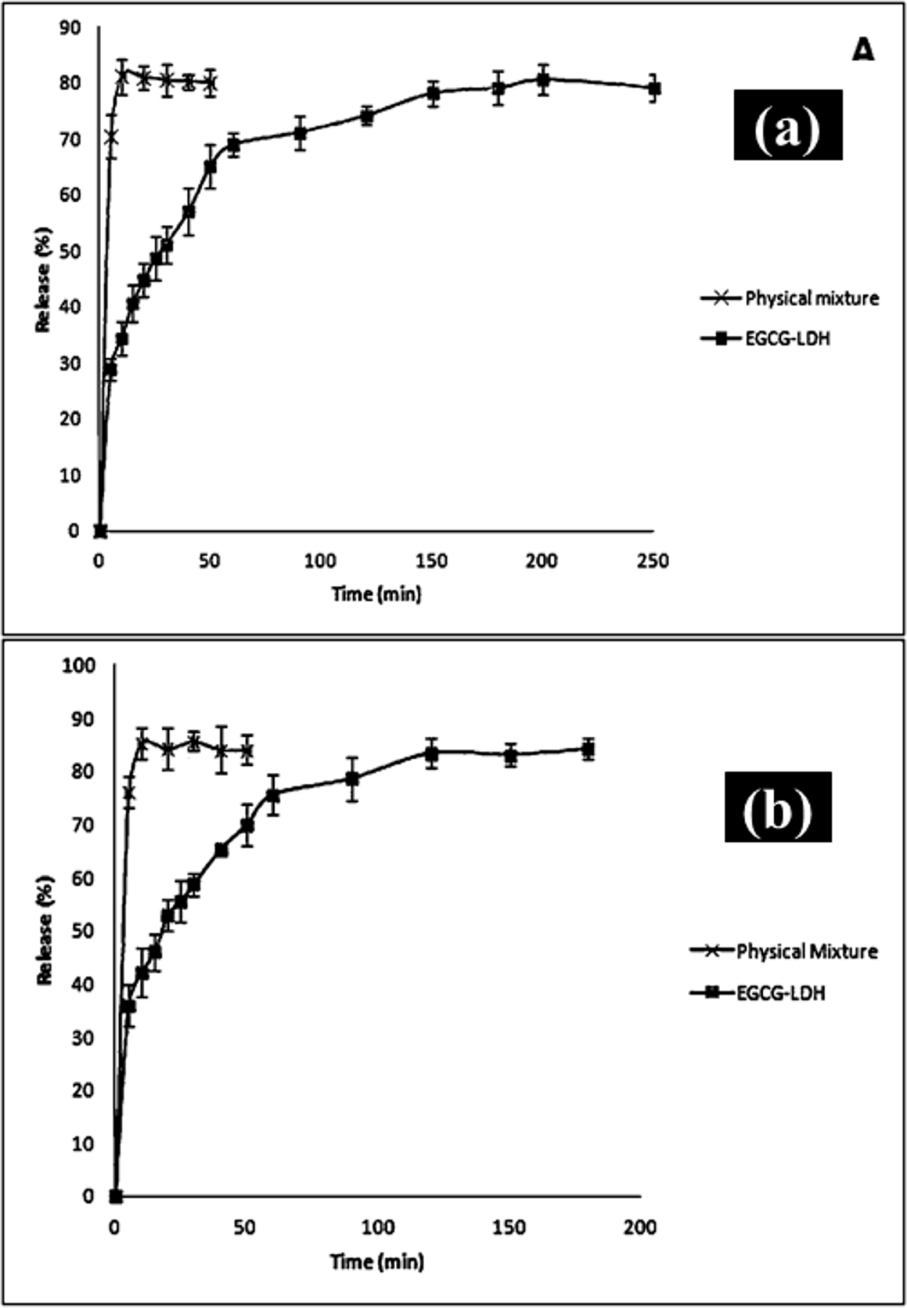
In-vitro Release profiles of EGCG from EGCG-LDH nanoparticle and physical mixture; at pH of a) 7.45 and b) 4.25.

The release profile curves for the EGCG from EGCG-LDH and the physical mixture (EGCG with LDHs) in buffer aqueous solutions at pH 4.25 and 7.45 were shown in Fig 9A and 9B. For the physical mixtures, more than 70% of total drug content was immediately released within the first 5 minutes. At pH 4.25 and 7.45, the release rate of EGCG from the physical mixture reached 84.08% and 80.07%, respectively. Evidently, physical mixtures of drugs and LDHs had an indiscernible sustained-release profile both in solutions at pH 4.25 and 7.45; while, EGCG intercalated LDHs presented a gradual release of the drug anions as a function of time both in solutions at pH 4.25 and 7.45.

In detail, the EGCG-LDH in pH 4.25 (see Fig 9B) showed two steps, with a burst release at the beginning of test (36% in first 5 min) followed by a relatively slow one (49% in 175 min). The burst release phenomenon was probably due to the release of the EGCG anions adsorbed in the LDH surface. After 180n min, 84.22% of the EGCG was released and the process reached the equilibrium. The destruction of LDH layers speeded up in the acidic medium, and as a result, more intercalated EGCG anions were released. Subsequently, by release of hydroxyl groups from the layers, the pH of the solution increased. In addition, the increase in the pH values resulted in the decrease of the EGCG release rate.

EGCG-LDH in pH 7.45 showed a similar release pattern (Fig 9A), with the released amount of 29% (in the first 5 min) during the burst and 79% (in 195 min) at the equilibrium state. Interestingly, the release of EGCG became much lower and sustained compared with pH 4.25. The total amount of released drug in pH 4.25 was greater than that of released drug in pH 7.45 due to acidic attack. It should be mentioned that the initial release rate of EGCG during the first 5 min at pH value of 4.25 was much faster than that in the other systems at pH of 7.45.

To explain the decrease of the release rate, some facts and details may be considered. The LDH is more stable and resistant at and above pH 7, and as a result, slow release process may be explicated based on the ion-exchange process between the interlayer anions and buffer anions. When small chemical species (chlorine and phosphates in the buffer) exchange bigger molecules (EGCG), a consequent decrease of the interlayer distance occurs. Consequently, a smaller (external part of the crystal) and a larger (internal part) interlayer distance co-exist in the same crystal and a phase boundary between two phases forms. As the exchange process proceeds, the boundary moves towards the central part of the crystal, and the drug release rate gradually decreases. Besides, at pH 7.4, two anions, H_2_PO4^−^ and HPO_4_^2−^, are generated by decomposition of the H_3_PO_4_, in which both can be intercalated into LDH inter layers [20], [21]. In our case, the acidic anions, once exchanged, reacted with the hydroxyls of the layer and might give grafting reactions. Therefore, phosphates were no longer exchangeable and could block exit of EGCG.

When the release process was finished, we found out that the EGCG anions could not entirely be exchanged. This was most likely due to the characteristic of ion-exchange reaction in these systems [22], [23]. Both the burst effect and sustained-release behavior are important in therapeutic treatments, because the early drug concentration in plasma provides an effective therapeutic dose; while the subsequent sustained release keeps this efficient dose over an extended period of time.

### The kinetic models

**Fig 10:**
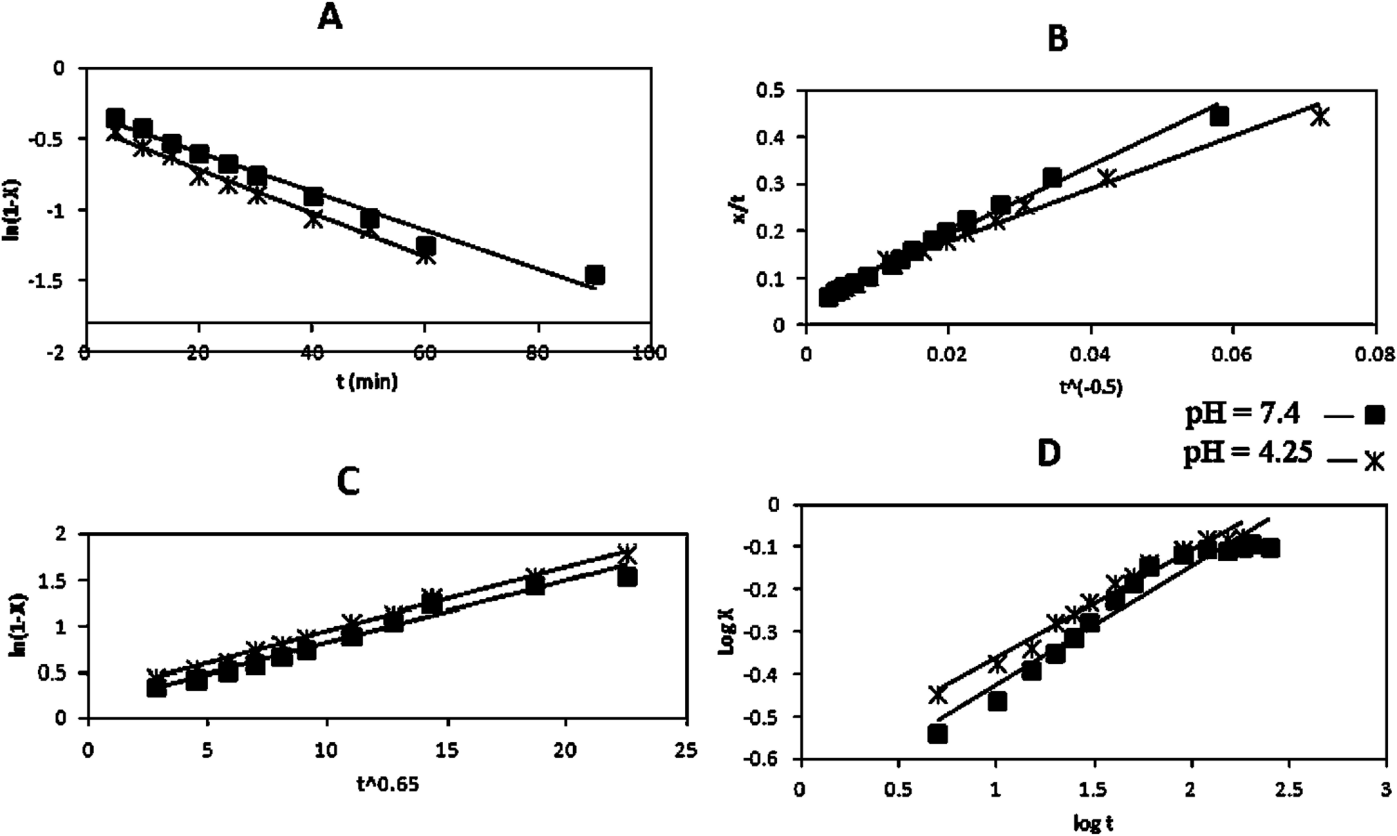
Plots of kinetic equations of (A) first-orderkinetic model, (B) parabolic diffusion model,(C) Bhaskar equation and (D) modified Freundlich model for the release of EGCG from EGCG-LDH nanoparticle at pH of 7.4 and 4.25.

To study the release mechanism of the EGCG from the LDH, four widely accepted kinetic models were used to fit the data. The results were shown in Fig 10.

The first-order kinetic model defines the release process that depends on the dissolution of the drug [24]:

$$\ln(\frac{\mathrm{Mt}}{M0})=-k1t$$

The parabolic diffusion model explains diffusion-controlled step in clays:

$$\frac{\left(1-(\frac{\mathrm{Mt}}{M0})\right)}{t}=-{\mathrm{kpt}}^{0.5}+m$$

In order to define the drug diffusion through the resins and inorganic materials the Bhaskar equation was used [25]:

$$\ln(\frac{\mathrm{Mt}}{M0})=-{\mathrm{kBt}}^{0.65}$$

The modified Freundlich model was used to understand the ion exchange properties of the system:

$$\frac{M0-\mathrm{Mt}}{M0}={\mathrm{kFt}}^{n}$$

Where, M_0_ and M_t_ are the drug concentration in hybrids at time 0 and t, respectively, the release rate constant, k, corresponds to the slope, and m and n the constants whose chemical significance is not clearly determined Based on the kinetics models, The two corresponding parameters obtained from the fittings (R^2^ and rate coefficient) were summarized in Table 3. The modified Freundlich model gave r^2^ ≤ 0.98 for the release profiles of EGCG-LDH at pH of 4.25 and 7.45, suggested that this model was not suitable to explain the release behavior of the EGCG-LDH. However, as shown in Table 3, the Bhaskar equation and parabolic diffusion model could explain the release processes of EGCG-LDH at pH of 4.25 and 7.45 by providing even reasonable r^2^ values more than 0.991. It was given that the parabolic diffusion model described the release process which was controlled by a diffusion process such as intraparticle diffusion, and the Bhaskar equation demonstrated the release profile where the diffusion through the particle was the rate limiting step. Diffusion of EGCG ions from the interlayer space of LDH sheets to medium became the rate-determining step, corresponding to the subsequent slow release period.

**Table 3:** Release rate constants and R^2^ coefficients obtained from release data fitting analyses based on kinetic equations.

	pH = 4.25	pH = 7.45		
Kinetic equation	R^2^	K	R^2^	K
**First order**	0.9883	0.015	0.9742	0.013
**Parabolic diffusion**	0.9832	0.5647	0.9911	0.731
**Bhaskar equation**	0.9959	0.07	0.9778	0.067
**Modified freundlich model**	0.9778	0.25	0.9407	0.27

## Conclusion

We conducted this study to show the efficacy of nanotechnology to improve the chemotherapeutic effectiveness of EGCG against PC3 cells. EGCG-LDH nanohybrid was successfully synthesized by by co-precipitation (LDH-NO3) and ion-exchange (LDH-EGCG) methods. The resulting nanohybrid exhibited more than 5-fold dose advantage over non-encapsulated EGCG. Elevated levels of apoptosis were observed in EGCG-LDH nanohybrids compared to plain EGCG and LDH at the same concentrations. Overall, In vitro release studies showed a two-step release, with observing a burst phenomenon at the beginning of the release test. Comparing the release dynamics with four kinetic models indicated the release of EGCG anions can be described by diffusion through the particle via ion exchange. Based on these findings, Ca/Al-LDH can be considered as an effective inorganic host matrix for the delivery of EGCG to PC3 cells with controlled release properties.
